# Targeting Glucose Metabolism of Cancer Cells with Dichloroacetate to Radiosensitize High-Grade Gliomas

**DOI:** 10.3390/ijms22147265

**Published:** 2021-07-06

**Authors:** Kristina M. Cook, Han Shen, Kelly J. McKelvey, Harriet E. Gee, Eric Hau

**Affiliations:** 1 Charles Perkins Centre, Faculty of Medicine and Health, University of Sydney, Sydney 2006, Australia; han.shen@sydney.edu.au (H.S.); kelly.mckelvey@sydney.edu.au (K.J.M.); harriet.gee@health.nsw.gov.au (H.E.G.); eric.hau@health.nsw.gov.au (E.H.); 2 Translational Radiation Biology and Oncology Laboratory, Centre for Cancer Research, Westmead Institute for Medical Research, Westmead 2145, Australia; 3 Bill Walsh Translational Cancer Research Laboratory, Kolling Institute, Faculty of Medicine and Health, University of Sydney, St. Leonards 2065, Australia; 4 Sydney West Radiation Oncology Network, University of Sydney, Sydney 2006, Australia; 5 Children’s Medical Research Institute, Westmead 2145, Australia

**Keywords:** radiotherapy, cancer metabolism, high-grade gliomas, glycolysis, dichloroacetate, hypoxia, radioresistance

## Abstract

As the cornerstone of high-grade glioma (HGG) treatment, radiotherapy temporarily controls tumor cells via inducing oxidative stress and subsequent DNA breaks. However, almost all HGGs recur within months. Therefore, it is important to understand the underlying mechanisms of radioresistance, so that novel strategies can be developed to improve the effectiveness of radiotherapy. While currently poorly understood, radioresistance appears to be predominantly driven by altered metabolism and hypoxia. Glucose is a central macronutrient, and its metabolism is rewired in HGG cells, increasing glycolytic flux to produce energy and essential metabolic intermediates, known as the Warburg effect. This altered metabolism in HGG cells not only supports cell proliferation and invasiveness, but it also contributes significantly to radioresistance. Several metabolic drugs have been used as a novel approach to improve the radiosensitivity of HGGs, including dichloroacetate (DCA), a small molecule used to treat children with congenital mitochondrial disorders. DCA reverses the Warburg effect by inhibiting pyruvate dehydrogenase kinases, which subsequently activates mitochondrial oxidative phosphorylation at the expense of glycolysis. This effect is thought to block the growth advantage of HGGs and improve the radiosensitivity of HGG cells. This review highlights the main features of altered glucose metabolism in HGG cells as a contributor to radioresistance and describes the mechanism of action of DCA. Furthermore, we will summarize recent advances in DCA’s pre-clinical and clinical studies as a radiosensitizer and address how these scientific findings can be translated into clinical practice to improve the management of HGG patients.

## 1. Introduction

High-grade gliomas (HGGs) are one of the deadliest and hardest to treat cancers. HGGs are fast-growing glial cell tumors found in the brain and spinal cord. In this review, we will largely be focusing on two types of HGGs: high-grade adult gliomas including glioblastoma multiforme, and pediatric diffuse pontine intrinsic gliomas (DIPG). Despite recent advances in multimodality treatment [1,2,3], their prognosis remains poor, and treatment is still challenging.

Deregulated metabolism is a universal hallmark of cancer. Tumor cells take up and metabolize nutrients such as glucose and glutamine to support key energetic processes, which are actively driven by oncogenes and the tumor microenvironment. This was initially thought to be due to the development of an uncontrolled cellular mass, leading to poor vascularization of the tumor and reduced oxygen supply. However, Otto Warburg observed that glucose was fermented via the glycolytic pathway even in the presence of ample oxygen, which was later referred to as aerobic glycolysis (or the Warburg effect) [4]. Like many other solid malignant tumors, HGGs preferentially use aerobic glycolysis to uptake and convert glucose into lactate. This altered glucose metabolism not only enables tumor cells to use glucose-derived carbons for the synthesis of essential cellular ingredients, but it also rapidly provides ATP to fuel cellular activities. In addition, this metabolic shift contributes significantly to treatment resistance including resistance to radiotherapy (RT) [5]. Thus, targeting these abnormal metabolic pathways could be employed as a novel approach to improving the radiosensitivity of HGGs.

**Figure 1:**
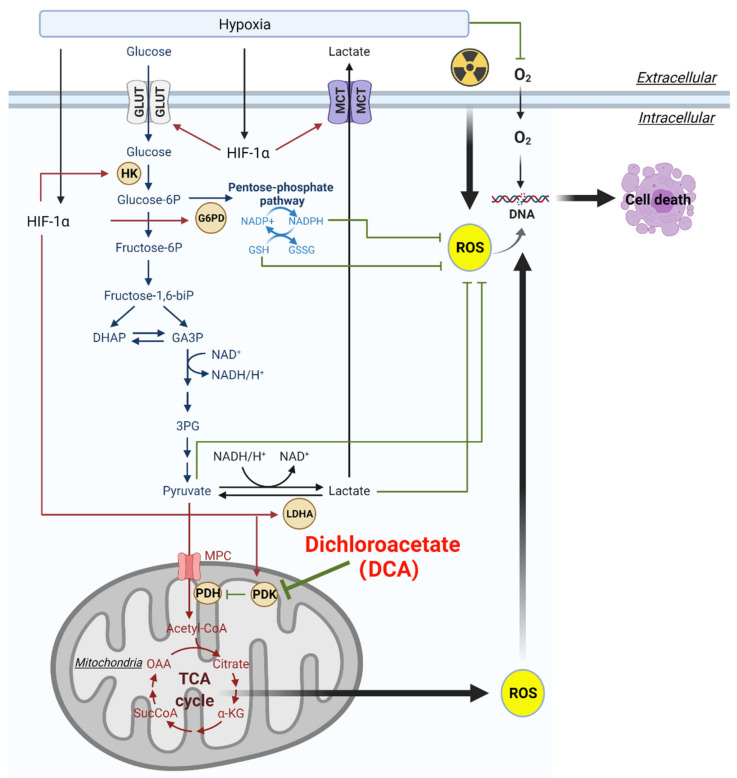
Hypoxia can regulate glycolysis, lactate, and pentose phosphate pathways in radioresistance. Dichloroacetate (DCA) has the potential to overcome radioresistance by targeting pyruvate dehydrogenase kinases (PDK). G6PD, glucose-6-phosphate dehydrogenase; GLUT, glucose transporters; GSH, glutathione; GSSG, glutathione disulfide; HIF-1, hypoxia inducible factor-1; HK, hexokinase; LDHA, lactate dehydrogenase A; MCT, monocarboxylate transporter; MPC, mitochondrial pyruvate carrier; PDH, pyruvate dehydrogenase; PDK, pyruvate dehydrogenase kinase; ROS, reactive oxygen species. Figure created with BioRender.com (accessed on 3 June 2021).

**Figure 2:**
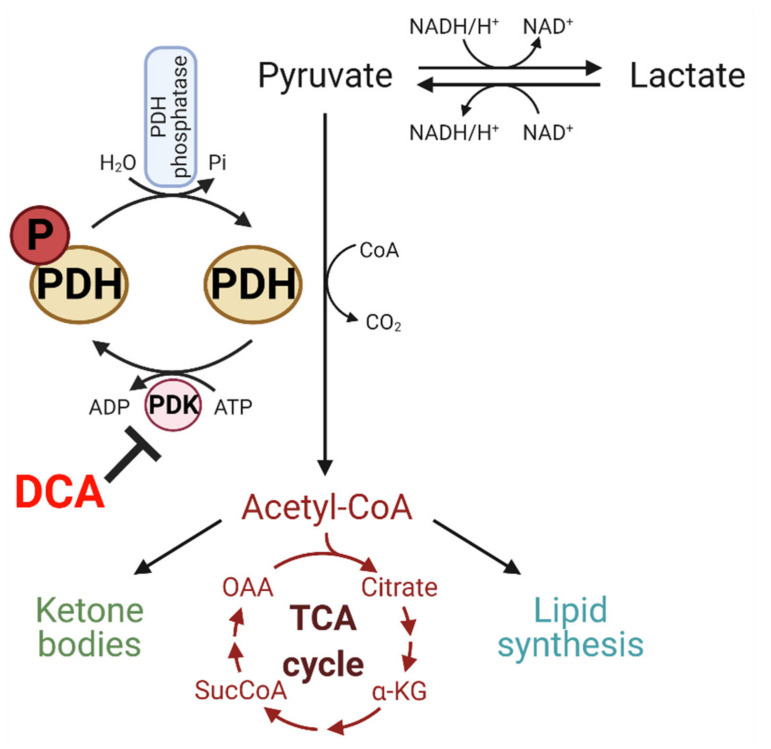
Mechanism of action for DCA. DCA inhibits pyruvate dehydrogenase kinases (PDK), increasing PDH activity and the conversion of pyruvate to acetyl-CoA. This has the effect of activating mitochondrial oxidative phosphorylation at the expense of glycolysis. Abbreviations: SucCoA (succinyl-coenzyme A), OAA (oxaloacetic acid/oxaloacetate), α-KG (α-ketoglutarate). Figure created with BioRender.com (accessed on 3 June 2021).

Deregulated glucose metabolism can be therapeutically targeted at different levels, from the master regulators that control glucose metabolism (e.g., hypoxia inducible factor [HIF]-1α, c-Myc) to key enzymes controlling rate-limiting steps in glycolytic pathways. One promising target is the pyruvate dehydrogenase kinase (PDK)/pyruvate dehydrogenase (PDH) axis. The inhibition of PDH activity by PDK is associated with many disorders including cancer. Dichloroacetate (DCA) is an inhibitor of PDK with the capability of maintaining PDH in its unphosphorylated active form (Figures 1 and 2). It was originally used as an experimental drug to treat lactic acidosis induced by congenital mitochondrial disorders and has recently been repurposed as an anticancer drug [6,7,8,9,10,11]. As a water-soluble small molecule, it can be rapidly absorbed with almost 100% bioavailability, and it readily crosses the blood–brain barrier, making it an excellent candidate for managing patients with brain cancers. In addition, the toxicity from DCA treatment is generally limited to a reversible peripheral neuropathy, but the same dose that causes neuropathy in adults with mitochondrial deficiency has been safely given to children with congenital mitochondrial diseases for many years [12]. These findings suggest that DCA may be most suitable for targeting pediatric brain tumors.

In this review, we will discuss the tumor microenvironment and altered glucose metabolism in HGGs. We will review DCA mechanisms of action and how this impacts tumor metabolism, followed by a summary of recent animal and human trials of DCA in HGGs in combination with radiotherapy (RT). The potential to translate these scientific findings into clinical practice is also discussed with the goal of improving the management of patients with HGG.

## 2. Aberrant Glucose Metabolism and Warburg Effect in HGGs

Like most cancer cells, HGG cells have altered cellular metabolism and are capable of shifting their primary metabolic pathways in response to nutrient availability in the surrounding microenvironment [13]. HGG cells rapidly uptake glucose from the surrounding microenvironment, and many HGGs show increased glycolysis as compared to surrounding brain parenchymal cells [14]. However, glucose is also the primary metabolic substrate in the healthy brain, and up to 35–40% of recurrent HGGs do not show higher positron emission tomography (PET) uptake of the glucose analogue fluorodeoxyglucose, as compared to surrounding brain tissue [15,16]. While HGG cells use glucose as a primary metabolic substrate, they can also use alternative metabolic substrates, such as fatty acids, amino acids, ketone bodies, and lactate [17]. There can also be intratumoral metabolic heterogeneity. For example, glioma stem cells (GSCs) have lower levels of glycolysis and lactate production compared to differentiated glioma cells, and GSCs are thought to rely primarily on oxidative phosphorylation (OXPHOS) [18]. GSCs are known for radioresistance, and this characteristic correlates with a higher mitochondrial reserve capacity [18].

Glucose metabolism, or glycolysis, converts one molecule of glucose into two molecules each of pyruvate, ATP, and NADH (Figure 1). There are two possible fates for pyruvate: (1) to be oxidized into acetyl-coenzyme A (acetyl-CoA), which enters the tricarboxylic acid (TCA) cycle for OXPHOS, or (2) pyruvate can be converted into lactate, which in normal cells tends to occur in low-oxygen conditions. In HGG cells, glucose is at least partially metabolized to lactate through the Warburg Effect, which refers to the non-oxidative metabolism of glucose even in the presence of sufficient oxygen. Pyruvate kinase muscle isozyme 2 (PKM2), an isoform of pyruvate kinase, is required for tumor cells to engage glycolysis and produce lactate, rather than send pyruvate through to the TCA cycle [19].

The Warburg effect can result in decreased pyruvate entering the mitochondria, decreasing OXPHOS. Glycolysis only yields 2 molecules of ATP from glucose, while glycolysis combined with OXPHOS yields up to 38 molecules of ATP, leading to the question of why cells would utilize metabolic pathways with a lower energy yield. It was initially believed that the Warburg effect was occurring due to defective mitochondria, but studies have shown that OXPHOS increases in HGG cells, indicating the presence of functioning mitochondria [20]. It is now thought that the Warburg effect gives HGG cells an advantage by supporting rapid cell proliferation and survival [21]. Glucose-derived carbons are used by cells to build metabolic intermediates for the biosynthesis of DNA, RNA, lipids, and proteins. Furthermore, the generation of intracellular lactate produces NAD+, which supports ongoing glycolysis and maintains redox balance. Secreted lactate creates an acidic tumor microenvironment, which has been shown to enhance tumor invasion [22] and immune evasion [23] of HGG cells. Lactate also alters the tumor microenvironment and enables tumors to adapt to hypoxia [22,23]. An increased rate of glycolysis in HGG cells can also help make up for the low rate of ATP synthesis as compared to OXPHOS; as while glycolysis is less bioenergetically efficient in generating ATP as compared to OXPHOS, the rate of glycolysis can be up to 100 times faster [24]. Thus, the Warburg effect is advantageous for HGG growth and invasion.

Along with upregulated glycolysis, HGG cells are still capable of utilizing glucose coupled with OXPHOS [20]. Recent studies of a patient-derived xenograft mouse model bearing orthotopic HGGs demonstrated that the classic bioenergetic adaptation described by Warburg may not be always present in HGGs. It has been reported that OXPHOS appears to be more important for glucose metabolism and chemo/radioresistance [20,25]. Indeed, HGGs, even within the same tumor, are inherently heterogeneous with histological and biological differences, varying degrees of immune infiltrates, and distinct molecular subtypes [26,27,28]. The heterogeneity in cancer cell metabolism and mitochondrial DNA content in HGG tumors and cell lines is not surprising [11,29,30,31,32]. While hierarchical clustering of gene expression data sets for metabolic pathways of 48 HGG primary cell lines showed no difference in glucose and fatty acid oxidation pathways among HGG subtypes [11], a larger cohort of 498 HGG IDH (isocitrate dehydrogenase) wildtype tumors suggests that mesenchymal tumors predominantly utilize glycolysis [29] and fatty acid oxidation (FAO) [31], while other subtypes are more variable [29]. Mitochondrial and glycolytic ATP production was observed to be a 1:1 ratio of OXPHOS to glycolysis among some individual primary GSC lines, while others heavily favored glycolysis and lactate production and are therefore more responsive to glycolytic inhibition [29]. FAO-dependent OXPHOS in HGGs can range from 25 to 60% [31], showing further plasticity and adaptability of HGG to nutrient-deprived microenvironments.

## 3. Hypoxia and Activation of HIF Can Alter Glucose Metabolism in HGGs

Oxygen is required for oxidative metabolism, and hypoxia is common in HGGs [33,34]. Glioblastoma (GBM; WHO grade IV gliomas) is defined by a hypoxic and necrotic core, encircled by a ‘pseudopalisading zone’ where cells escape hypoxia by invading healthy tissue [35]. The diffuse midline gliomas, such as DIPG, have also been found to be hypo-perfused, indicating these types of tumors are also likely to be hypoxic [36], though more studies are needed to understand the role of hypoxia in these tumors. Pseudopalisading cells contribute directly to the malignant behavior of HGGs by acting as an invasive front of the tumor by migrating away from hypoxia [35]. Hypoxic tumor cells survive these harsh conditions by activating the transcription factor HIF, which is composed of an oxygen-sensitive HIF-1α subunit and a constitutively produced HIF-1β subunit. HIF is currently estimated to be capable of regulating >1000 genes in humans, including many key metabolic enzymes [37]. Activation of HIF is therefore associated with metabolic reprogramming (Figure 1). HIF can upregulate the expression of genes encoding glucose transporters and nearly every glycolytic enzyme, including hexokinases, phosphofructokinase 1, phosphoglycerate kinase 1, and pyruvate kinase M2, and thereby leading to an increase in glycolysis [37]. HIF also uncouples glycolysis and OXPHOS by expressing PDK [38], which phosphorylates and inactivates PDH. Subsequently, the inhibition of PDH by PDK prevents the conversion of pyruvate to acetyl-CoA by blocking entry into the TCA cycle. HIF also upregulates lactate dehydrogenase A (LDHA), which converts pyruvate to lactate, and the lactate transporter monocarboxylate transporter 4 (MCT4), which exports lactate to the extracellular environment [39]. HIF increases expression of the BNIP3 gene, which promotes mitochondrial-selective autophagy, which reduces oxidative metabolism [40]. HIF also expresses many genes important for glioma progression, including those that regulate invasion and stem cell markers.

The Warburg effect can be partially driven by HIF-1α. Cancer cells have increased growth factor signaling, mutations (e.g., pVHL), and accumulation of TCA intermediates (succinate and fumarate), which increase and stabilize HIF-1α even in the presence of sufficient oxygen [41]. Furthermore, pathological stabilization of HIF-1α is facilitated by the accumulation of pyruvate and lactate that are generated in glycolysis, which prevent HIF-1α from being degraded [42]. Many studies have reported poor survival and prognoses in HGG patients with high levels of HIF-1α, PDKs, and tumor lactate concentrations [43,44], and inhibition of HIF-1α in hypoxia increases the radiosensitivity of HGGs [45].

As glycolysis increases and uncouples from the TCA cycle by producing lactate, glutamine metabolism is reprogrammed to top up and maintain flux of TCA intermediates. HIF activation assists switching from oxidative decarboxylation to reductive carboxylation of glutamine to maintain citrate and acetyl-CoA for lipid synthesis [46,47,48]. Hypoxic HGG cells are unable to survive if they are glutamine starved [47]. Hypoxia and subsequent HIF activation is therefore a critical tool for metabolic adaptations in HGG cells.

## 4. Radiotherapy and Radioresistance in HGGs

RT is the cornerstone of treatment for many types of cancer including HGGs. RT is used either as a palliative treatment for inoperable HGGs or following surgery to kill residual HGG cells in the tumor bed. The efficacy of RT primarily relies on its ability to damage the DNA of cancer cells and subsequently cause cell death [49]. Despite RT being one of the most effective therapies for HGG treatment, most HGGs inevitably relapse due to the intrinsic/acquired radioresistance of HGG cells, especially when GSCs and a ‘hypoxic niche’ are present [50]. Radioresistance is an evolutionary process in which intrinsically radioresistant cells are either ‘selected for’ by RT, or the surviving HGG cells develop acquired resistance due to genetic and/or metabolic changes induced by RT. This intricate process involves multiple mechanisms and remains to be fully elucidated. Several mechanisms have been proposed as important contributors to radioresistance, including (i) improved capability of DNA repair, (ii) cell cycle arrest at relatively radioresistant phases (namely G_0_ and late S-phase), (iii) alterations of gene expression and microenvironment which counteract the effect of RT, (iv) induction of autophagy in cancer cells as a self-protective mechanism, (v) generation of cancer stem cells (CSCs), and (vi) rewiring of metabolic pathways to be more radioresistant [51,52,53].

Most HGGs will relapse due to the presence of radioresistant GSCs, which are enriched in hypoxic regions of the tumor [54]. Oxygen is required to generate the DNA-damaging reactive oxygen species (ROS) and reactive nitrogen species (RNS) that act as the central mechanism through which external beam X-ray photon RT works, and hypoxia limits this process [55,56]. In addition, the HIF-mediated glycolytic phenotype has also been linked to the increased antioxidant capacity of tumor cells, which significantly blunts the efficacy of RT [57]. In a recent study investigating radioresistant mechanisms in DIPG, mutations in tumor protein (TP)53 have been identified as a main driver of radioresistance in patient-derived DIPG biopsies as well as their corresponding clinical cohort [58]. TP53 regulates glycolysis through several modes of action including suppression of the expression of glucose transporters, inhibition of rate-limiting glycolytic enzymes, reduction in the cellular excretion of lactate, and down-regulation of the protein kinase B (AKT)/mTOR [59] and nuclear factor (NF)-κB signaling pathways [60]. TP53 mutations can therefore lead to increased glycolysis. TP53 also regulates the expression of key glycolytic enzymes such as phosphoglycerate mutase and TP53-induced glycolysis and apoptosis regulator (TIGAR) [61].

RT induces changes in the tumor microenvironment and adjacent healthy brain parenchyma. These include changes in cellular bioenergetics, vascularity, and immune activation leading to altered drug metabolism, reduced therapeutic efficacy, and/or radioresistance [62]. Metabolic analyses using proton nuclear magnetic resonance spectroscopy of brain tissue pre- and post-irradiation with 20 Gy revealed a significant increase in energy carriers (ATP, GTP), reduced levels of antioxidants (GSH, ASC, NAD+) and TCA metabolites (citrate, succinate, fumarate), and increased tumor aggression [62]; though the effects were lesser with fractionated radiation (40 Gy/4 fractions vs. 20 Gy). These data highlight that dual targeting of cancer cell metabolism and RT may be a viable therapeutic strategy by utilizing a bioenergetic blockade to re-sensitize HGG to RT. Furthermore, metabolites may serve as biomarkers to identify which bioenergetic pathway/s to therapeutically target in an individual patient.

## 5. DCA Inhibits PDK and Has Potential to Modulate Glucose Metabolism

The PDK/PDH axis is considered the gatekeeper linking cytoplasmic glycolysis to the TCA cycle and OXPHOS in mitochondria (Figure 2). PDH is post-translationally regulated by reversible phosphorylation of the E1α subunit, with the phosphorylated form being catalytically inactive. Under normal physiological conditions, PDH is regulated by changes in PDK activity or expression, providing feedforward stimulation and feedback inhibition. Accumulation of the PDH reaction products (ATP, NADH, and acetyl-CoA) promotes PDK activity and inhibits PDH via phosphorylation at one or more serine residues on the E1α subunit, while stimulating PDK activity. In contrast, the substrates for the PDH reaction suppress PDK and restore PDH activity (ADP, NAD+, and pyruvate) [63].

DCA was identified as a metabolically active moiety of diisopropylammonium dichloroacetate back in 1970 [64]. Although DCA has been indicated for various disorders, it remains an experimental drug that is not approved by the Food and Drug Administration for any condition. DCA enters cells via the monocarboxylate transporters (MCT) and gains access to the mitochondrial matrix by the mitochondrial pyruvate transporters (MPC) [65]. DCA increases PDH activity by competitively binding to the same binding site of pyruvate at the PDK N-terminal regulatory R domain, leading to inhibition of PDK activity (Figure 2). An oral dose of DCA is rapidly absorbed and widely distributed within minutes of administration [65]. It readily crosses the blood–brain barrier and can be measured in cerebrospinal fluid. Blood lactate concentrations begin to fall within about 15–30 min following oral or parenteral dosing, so it can be used as a sensitive biomarker for DCA’s on-target action on PDH [65]. As discussed above, cancer cells require both energy and biomass, which can be achieved by upregulating glycolysis through the Warburg effect [66]. DCA reverses the Warburg effect in tumor cells by inhibiting PDKs, restoring PDH activity, boosting OXPHOS, and decreasing pyruvate and lactate levels, leading to decreased expression of HIF-1α [67].

## 6. Pre-Clinical Evidence of DCA as a Radiosensitizer in HGG Treatment

The relapse of HGGs following radiotherapy is due in part to intrinsic or acquired radioresistance mediated by hypoxia and deregulated glucose metabolism. DCA in this setting could represent a strategy to overcome tumor radioresistance by suppressing HIF-1α, altering glucose metabolism and inducing higher levels of ROS in tumor cells. Our own studies have demonstrated the efficacy of DCA to increase radiosensitivity in both adult and pediatric HGG pre-clinical models [10,32].

### 6.1. DCA and Glioma Stem Cells (GSCs)

There is a growing interest in targeting CSCs, which are one of the main drivers of radioresistance [68]. CSC/GSC are a distinct subpopulation within the tumor bulk. They possess a high capability to repair DNA damage, exhibit low levels of ROS, and proliferate slowly, which all potentially contribute to radioresistance. An extensive body of literature covers the metabolic phenotype of CSCs, which seem to differ from differentiated cancer cells and may represent a novel therapeutic target [69]. DCA is proposed to both increase stem cell differentiation and increase radiosensitivity in glioblastoma [70].

### 6.2. Efficacy of DCA Combined with Radiotherapy

Despite the metabolic heterogeneity of HGGs, the cell survival/viability (in vitro) IC_50_ of DCA is comparable among HGG stem cells (15–40 mM) [29], immortalized human (20–28 mM), and rodent glioma cell lines (27–28 mM) [11,71]. Although cell death HGG IC_50_ values are at millimolar levels, concentrations of DCA as low as 0.1 and 1 mM still induce radiosensitization, decrease clonogenic survival, and increase sub-G1 specific apoptosis in human primary glioblastoma cells [72]. DCA appears to cause in vitro radiosensitization through the following mechanisms: reducing mitochondrial reserve capacity, increasing ROS-induced DNA damage, and inducing cell cycle arrest and autophagy, which ultimately leads to cell death [10,11]. Using the intracranial and in vivo human HGG U87-MG model, the combination of DCA and fractionated RT significantly reduced tumor Ki-67 proliferation and improved median survival by a modest 13%, when compared to RT alone [10].

### 6.3. Efficacy of DCA/Radiotherapy Combined with Chemotherapy or Metabolic Drugs

DCA may also have anti-cancer potential when used in combination with radiotherapy and other drugs. In glioblastoma, standard treatment comprises fractionated radiation with concomitant and adjuvant temozolomide chemotherapy [2]. Temozolomide inhibits the repair of RT-induced DNA damage; however, its therapeutic efficacy is limited in patients with an un-methylated promoter for the methylguanine-DNA-methyltransferase (MGMT) gene, which encodes a DNA repair enzyme affording chemoresistance. Intracranial 9L tumor-bearing rats treated with oral gavage or wafer DCA (50% DCA by weight incorporated into a biodegradable polymer pCPP:SA wafer), combined with temozolomide and RT showed increased median survival by 91% and 62% compared to control and 80 mg/kg/d oral gavage DCA (day 0 to end of life), respectively, though not when compared to temozolomide/RT [71].

HGGs are characterized by VEGF-mediated vascularization leading to highly disorganized tumor vasculature, focal regions of tumor hypoxia, and impaired oxygen-dependent RT-induced DNA damage. In glioblastoma patients, bevacizumab, an anti-angiogenic monoclonal antibody that targets VEGF-A, has been explored in conjunction with standard therapy to reduce tumor vascularization and restore normal vessel architecture [73,74]. Unfortunately, this approach led to a more hypoxic, glycolytic, and invasive tumor phenotype, further limiting RT efficacy. Interestingly, DCA also demonstrates anti-angiogenic activity by increasing microvascular apoptosis [75]. Kumar et al. demonstrated that bevacizumab in combination with DCA significantly reduces tumor growth using in vitro spheroid models and in vivo U87-MG xenograft models [76]. Whether a tri-modality approach of DCA, bevacizumab, and RT is viable has yet to be explored.

Dual-targeting of HGG bioenergetics using DCA in combination with other metabolic blockades has also been trialed to circumvent the plasticity HGG cells possess in adapting their ‘primary’ metabolic pathways following chemoradiation [77]. DCA combined with metformin or phenformin, which are biguanides that lower blood glucose levels via a blockade of hepatic gluconeogenesis, inhibits HGG cell proliferation and increases apoptosis in vitro [77,78,79], which reduces tumor growth and increases median survival in vivo [79]. DCA and metformin have been combined with RT to radiosensitize tumors, and the triple combination increased median survival by 26% in human DIPG007 tumor-bearing mice compared to RT alone [32]. Other studies using syngeneic murine models of HGG combined a glycolytic and FAO inhibitor and showed modest benefit in median survival of tumor-bearing animals, but it supported long-term survival when used in combination with standard chemoradiation [11,31].

In addition to the direct actions of DCA and other metabolic inhibitors on cancer cells, these drugs alter the tumor immune response via shifts in cytokine profiles, differential transcriptional regulation, abrogation of the immunosuppressive network, and reduction in lactate [62,80]. Given that T cells are absent in immunocompromised human HGG xenograft models and are key drivers of the immune response, immunocompetent models may be required to demonstrate the complete picture of therapeutic efficacy of radiosensitization through inhibition of glycolysis.

### 6.4. Radiosensitivity Induced by DCA Varies Depending on In Vitro and In Vivo Models

While DCA leads to radiosensitization of HGG cells in vitro, these results have not always been able to be replicated in vivo. In vitro DCA/ranolazine significantly reduced clonogenicity of HGG cells and induced ROS, DNA damage, autophagy, and apoptosis. Despite these initial results, only a modest ~20–30% increase in in vivo median survival was seen in orthotopic syngeneic murine HGG models [11]. The contrasting in vitro and in vivo results are often suggested to be due to the changes induced by the addition of a complex tumor microenvironment within a whole organism. However, there are also differences between the time-course and pharmacokinetics of in vitro versus in vivo methodologies, which are important for DCA, with its complex kinetics. In vitro assays typically conclude 72 h post-treatment without drug wash out, while in vivo murine models are considerably longer, with DCA undergoing rapid metabolic clearance from the body.

Zwicker et al. demonstrated DCA can induce radiosensitization in LN18 glioblastoma and WIDR colorectal cells in vitro, but not in vivo [72]. Histopathological analyses revealed that DCA reduced Ki67 proliferation and induced apoptosis at day 4 (last day of treatment); however, by day 20 this reverted to increased Ki67 proliferation and ~40% increase in tumor hypoxia [72]. Cellular and metabolic changes associated with DCA revert upon cessation of the treatment; thus, DCA-related tumor control and radiosensitization requires concomitant DCA/RT and then continued adjuvant DCA administration. The requirement of long-term administration of DCA to achieve therapeutic efficacy is further evidenced in a study by Shen et al., wherein extending DCA treatment to ‘end of life’ (after concomitant DCA/RT treatment) increased in vivo U87-MG glioblastoma median survival by ~50%, when compared to untreated tumor-bearing animals [10].

In a rat allograft 9 L glioblastoma model, biodegradable wafers were utilized to deliver high-dose DCA within millimeters of the tumor [71]. These wafers have a slow, dual phase release with the majority of drug released in the first 10 h, and the remainder over the next 6–8 weeks as the wafer is absorbed, enabling a form of long-term administration [81].

### 6.5. DCA Efficacy and microRNAs

Finally, the response of cells to DCA may be influenced by post-transcriptional regulation of key metabolism gene expression, such as via microRNAs. From bioinformatic databases, miR-144 is predicted to target PDK1, TIGAR, IDH1, and IDH2 [82], which are involved in cellular bioenergetic pathways. Expression of miR-144 was down-regulated in both HGG cells lines and 19/24 glioblastoma tumors, compared to normal human astrocytes [82]. Overexpression of miR-144 decreased PDK1 protein levels, impaired energy metabolism by decreasing glycolytic flux and capacity in U87-MG, and reduced mitochondrial and ATP-coupled respiration in DBTRG cells [82]. While U87-MG cells are widely used in HGG research, they have low mRNA expression of PDK1, compared to up-regulated expression in DBTRG cells and patient-derived HGG cells, and these differences may explain some of the different results seen with miR-144 [82]. Ionizing radiation is known to alter expression of miRNAs [83], which may underlie DCA radiosensitization. Alternatively, it may hinder it due to the role of miRNAs in the cellular stress response and stimulation of DNA repair mechanisms [83].

## 7. Clinical Trials of DCA in Cancer and as an Anti-HGG Drug

There are five published reports of phase I/II clinical trials using DCA for the treatment of cancer patients (Table 1). Two trials focused on brain tumors (5 and 15 patients) [75,84], one in patients with non-small cell lung (NSCLC) and breast cancer (7 patients) [85], one on myeloma patients [86], and the largest trial (23 patients) included a wide range of solid tumors [87]. In the phase 2 study by Garon et al., six patients with stage IIIB/IV NSCLC and one patient with breast cancer were enrolled [85]. Two patients died within a week of DCA, with one due to pulmonary embolism and another possibly due to cerebrovascular accident. Its relationship to DCA is uncertain, and the patients in this study may not have received therapeutic levels to achieve efficacy. Mean and median plasma DCA levels need to exceed the Ki of DCA of ~25 μg/mL (~0.2 mmol/L) for PDK2, the most ubiquitously expressed PDK isoform and the most sensitive to DCA inhibition [88], and this can take months to achieve [75]. However, due to the two deaths and lack of apparent clinical activity, the data safety and monitoring committee closed the study.

Long-term administration of millimolar concentrations of DCA presents several challenges in the clinical setting. A pilot study in glioblastoma patients [75] indicated that it could take months for serum DCA concentrations to reach hypothetically effective levels. In the Michelakis et al. trial [75], three glioblastoma patients who had refractory tumors were treated with DCA alone, and an additional two newly diagnosed glioblastoma patients received DCA in combination with standard radiation and temozolomide therapy after debulking surgery. Due to the small number of patients, conclusions about clinical efficacy cannot be established, but peripheral neuropathy was the only apparent toxicity. On the tissues derived from these patients, it was shown that DCA could decrease proliferation and reduce angiogenesis. In vitro and in vivo experiments showed that in both the glioblastoma cells and glioblastoma stem cells, DCA increased apoptosis and reactive oxygen species, while decreasing mitochondrial potential and HIF-1α.

In the largest clinical trial of DCA, Chu et al. [87] enrolled 24 patients with various malignancies on a starting dose of 6.25 mg/kg bd (twice daily). However, due to toxicities with a higher dose of 12.5 mg/kg bd, the maximum tolerated dose (MTD) was established at 6.25 mg/kg bd. Common toxicities of any grade included fatigue, neuropathy, anorexia, and nausea. There was a high variability of trough levels between patients, but there was an overall progressive increase in trough DCA levels with time. Of the seventeen patients evaluable for response, eight had stable disease. There were no partial or complete responses.

In a “3 + 3” study design in 15 patients (2 patients with metastatic brain tumors, 13 malignant glioma), DCA dosing was based on haplotype variation in glutathione transferase zeta 1/maleylacetoacetate isomerase (GSTZ1/MAAI), which participates in DCA and tyrosine catabolism [84]. Patients who have at least one wild-type haplotype for GSTZ1/MAAI metabolize DCA more rapidly than those who lack this haplotype. Patients deemed “fast” metabolizers continued on the “3 + 3” design, whilst those who were deemed slow metabolizers were administered 4 mg/kg/12 h. Eight evaluable patients had clinically and radiographically stable disease at the end of the fourth week of DCA. No patient was withdrawn due to toxicity of DCA and showed that oral DCA administered within the dose range established in metabolic disease is safe and well tolerated in adults.

Due to the potential impact of GSTZ1 genetics on DCA metabolism, an Australian study by Tian et al. [86] investigated oral DCA for 3 months with a loading dose in seven myeloma patients with 2–7 prior therapies. One patient responded, and two patients showed a partial response. The initial half-life of DCA was shorter in two patients, correlating with heterozygosity for the GSTZ1*A genotype. Another patient had a trough concentration 3-fold higher than others, which correlated with a low activity promoter genotype (-1002A, rs7160195). This corresponded with response but also with peripheral neuropathy.

It is worth noting that a reversible peripheral neuropathy was reported from DCA treatment in several species, including humans. However, recent clinical trials indicate that adults are considerably more susceptible to this adverse effect than children. A later study then evaluated the kinetics of DCA and found a striking age-dependent decrease in DCA in its plasma clearance and an increase in its plasma half-life in both murine models and human patients [89]. This age-dependent metabolism and elimination of DCA leads to less toxicity in children, indicating it may be a safer drug for treatment of pediatric patients with brain tumors.

All of the clinical trials of DCA are early-phase studies and, as such, conclusions about efficacy are limited. There does however appear to be at least some evidence of modest activity, and it can be generally safely delivered.

## 8. Conclusions and Future Directions

Targeting metabolism of HGG cells with DCA represents a new pharmacological approach to treat cancer. The ability of DCA to reverse the aberrant glucose metabolism has increased interest in this drug, which is already known for its anticancer properties. Recent evidence in vitro and in vivo confirms the capability of DCA to overcome radioresistance in several cancer types including HGGs. The conclusion from the clinical studies is that DCA is generally safe, though the effect of GSTZ1 genetics on pharmacokinetics, toxicities, and efficacy needs to be explored further. The very limited clinical data do support some clinical activity of DCA but are probably best trialed in cancer populations, in combination with other established anti-cancer therapies such as radiation therapy, and in those tumors which are not rapidly progressive due to the time required for DCA to achieve therapeutic levels in serum.

There is a growing number of pre-clinical studies designing and testing new formulations of DCA (reviewed by Tararanni and Piccoli [90]). The goal of these studies is to improve efficacy and delivery of DCA and analogues and reduce adverse effects. These novel, complex compounds containing DCA and other drugs seem to be more potent than the sodium salt of DCA in pre-clinical models, which warrants further clinical evaluation. Due to a lack of data from clinical trials investigating DCA as a radiosensitizing reagent, there is an unmet need to design further clinical studies investigating DCA as treatment for patients with HGG.

## Figures and Tables

**Table 1:** Summary of phase I/II clinical trials of DCA in cancer.

Clinical Trial	Description	Population Description	Main Conclusions	Ref.
Michelakis et al.	Small study of 49 freshly isolated glioblastoma samples and 5 patients with glioblastoma	5 patients with glioblastoma	Indications of clinical efficacy were present at a dose that did not cause peripheral neuropathy and at serum concentrations of DCA sufficient to inhibit the target enzyme of DCA	[75]
Garon et al., NCT01029925	Open label phase II trial	6 patients with stage IIIB/IV non-small cell lung (NSCLC) and one patient with breast cancer	Firm conclusions regarding the association between these adverse events and DCA are unclear. Further development of DCA should be in patients with longer life expectancy, in whom sustained therapeutic levels can be achieved, and potentially in combination with cisplatin.	[85]
Tian et al.	Open label non randomized phase II trial	7 myeloma patients	Promoter *GSTZ1* polymorphisms may be important determinants of DCA concentrations and neuropathy during chronic treatment. Novel dosing regimens may be necessary to achieve effective DCA concentrations in cancer patients while avoiding neuropathy.	[86]
Dunbar et al., NCT01111097	Open-label single-arm phase 1 study	15 adults with recurrent WHO grade III–IV gliomas or brain metastases from a primary cancer outside the central nervous system	Chronic, oral DCA is feasible and well-tolerated in patients with recurrent malignant gliomas and other tumors metastatic to the brain. Genetic-based dosing is confirmed and should be incorporated into future trials of chronic DCA administration.	[84]
Chu et al.	Open-label phase 1 study	24 patients with advanced solid malignancies	Progressive increase in DCA trough levels and a trend towards decreased (18) F-FDG uptake with length of DCA therapy was observed. The recommended phase II dose of DCA is 6.25 mg/kg BID.	[87]

## Data Availability

Not applicable.
